# Identification of key control points in instrument reprocessing of hospital central sterile supply department and its association analysis with surgical site infection

**DOI:** 10.3389/fpubh.2026.1844524

**Published:** 2026-08-13

**Authors:** Xia Lin, Jie Wu

**Affiliations:** 1 Department of Central Sterile Supply, The People's Hospital of Liyang City, Liyang, Jiangsu, China; 2 Department of Hospital Infection Management, The People's Hospital of Liyang City, Liyang, Jiangsu, China

**Keywords:** central sterile supply department, instrument reprocessing, patient safety, quality control, sterilization, surgical site infection

## Abstract

**Background:**

The Central Sterile Supply Department (CSSD) plays a crucial role in preventing healthcare-associated infections through proper instrument reprocessing. However, critical control points in the reprocessing workflow and their direct association with surgical site infection (SSI) remain inadequately characterized.

**Objective:**

To identify key control points in CSSD instrument reprocessing and analyze their association with surgical site infection (SSI) rates, defined according to Centers for Disease Control and Prevention (CDC) criteria through a comprehensive retrospective analysis.

**Methods:**

A retrospective cohort study was conducted from January 2020 to December 2023 at a tertiary care hospital. We analyzed 15,847 surgical procedures and corresponding CSSD reprocessing records. Key control points were identified through process mapping, failure mode analysis, and statistical correlation with infection outcomes. Multivariate logistic regression was used to determine independent risk factors.

**Results:**

Seven critical control points were identified: pre-cleaning verification (OR: 2.34, 95% CI: 1.78–3.09), cleaning efficacy validation (OR: 3.12, 95% CI: 2.45–3.98), sterilization parameter monitoring (OR: 4.67, 95% CI: 3.89–5.61), sterile integrity maintenance (OR: 2.89, 95% CI: 2.21–3.78), staff competency verification (OR: 2.56, 95% CI: 1.99–3.29), equipment maintenance compliance (OR: 3.45, 95% CI: 2.67–4.46), and quality documentation completeness (OR: 1.98, 95% CI: 1.54–2.54). Overall SSI rate was 2.4% (380/15,847 procedures). Inadequate control at identified points increased infection risk by 180–367%.

**Conclusion:**

Seven key control points are significantly associated with SSI rates. Implementation of enhanced monitoring and control measures at these points may be associated with SSI and improve patient safety outcomes.

## 1 Introduction

Healthcare-associated infections (HAIs) represent one of the most significant challenges in modern healthcare systems, affecting millions of patients worldwide and imposing substantial economic burdens on healthcare institutions (1). Among various types of HAIs, surgical site infections (SSIs), constitute a major category that directly impacts patient outcomes, prolongs hospital stays, and increases healthcare costs (2). The prevention of SSI requires a multifaceted approach, with proper sterilization and reprocessing of surgical instruments serving as a cornerstone of infection control strategies.

The Central Sterile Supply Department (CSSD) functions as the critical hub for decontamination, cleaning, disinfection, sterilization, and distribution of reusable medical devices and surgical instruments throughout healthcare facilities (3). The complex processes undertaken within CSSD directly influence the safety and efficacy of surgical procedures by ensuring that all instruments are free from viable microorganisms and pyrogens. However, the intricate nature of instrument reprocessing workflows presents numerous opportunities for process failures, which may compromise sterility assurance and subsequently higher infection risks (4).

Current literature has extensively documented the importance of various components within the sterilization process, including cleaning protocols, sterilization parameters, packaging integrity, and storage conditions (5). However, there remains a critical gap in understanding which specific control points within the CSSD workflow have the most significant impact on surgery-related infection outcomes. Traditional approaches to CSSD quality management often employ broad-spectrum monitoring without prioritizing resources toward the most critical control points, potentially leading to inefficient resource allocation and suboptimal infection prevention outcomes (6).

The concept of critical control points (CCPs) has been successfully applied in various industries, particularly in food safety through Hazard Analysis and Critical Control Points (HACCP) systems (7). This systematic approach identifies specific points in a process where hazards can be prevented, eliminated, or reduced to acceptable levels through monitoring and control measures. The application of similar principles to CSSD operations could potentially revolutionize infection prevention strategies by focusing attention and resources on the most impactful process elements (8).

Recent advances in process monitoring technologies, data analytics, and quality management systems have created unprecedented opportunities to systematically analyze CSSD operations and their correlation with clinical outcomes (9). The integration of electronic monitoring systems, automated data collection, and advanced statistical analysis methods enables comprehensive evaluation of process performance and its relationship with patient safety indicators. This technological evolution provides the foundation for evidence-based identification of critical control points and development of targeted intervention strategies (10).

The economic implications of SSI extend far beyond immediate treatment costs, encompassing extended hospital stays, additional procedures, long-term complications, and potential legal liabilities (11). Conservative estimates suggest that each preventable SSI costs healthcare systems between $13,000 and $35,000, with more complex cases potentially exceeding $100,000 in additional expenses. Given the substantial financial impact, investments in optimizing CSSD operations through systematic identification and control of critical points present compelling opportunities for both improving patient outcomes and reducing healthcare costs (12).

Therefore, this retrospective study aims to systematically identify key control points within CSSD instrument reprocessing workflows and quantitatively analyze their association with surgery-related infection rates. By employing comprehensive process mapping, statistical analysis, and multivariate modeling techniques, we seek to provide evidence-based insights that can guide targeted quality improvement initiatives and enhance patient safety outcomes. The findings from this research are expected to contribute to the development of risk-based CSSD management strategies and inform evidence-based guidelines for optimizing sterilization processes in healthcare settings.

## 2 Methods

### 2.1 Study design and setting

This retrospective cohort study was conducted at a 1,200-bed tertiary care academic medical center serving a catchment population of approximately 2.5 million people. The institution performs over 25,000 surgical procedures annually across multiple specialties, including general surgery, orthopedics, cardiovascular surgery, neurosurgery, and minimally invasive procedures. The study protocol was approved by the Institutional Review Board with a waiver of informed consent due to the retrospective nature of the research and use of de-identified data.

The hospital’s CSSD operates as a centralized facility processing approximately 180,000 instrument sets annually, serving 32 operating rooms, multiple procedure areas, and various clinical departments. The department employs 45 full-time equivalent staff members working across three shifts to ensure continuous operation. All reprocessing activities follow standardized protocols based on national guidelines and manufacturer instructions, with comprehensive documentation maintained through an integrated quality management system.

### 2.2 Study population and inclusion criteria

The study population comprised all patients undergoing surgical procedures between January 1, 2020, and December 31, 2023. Inclusion criteria were: (1) patients aged 18 years or older; (2) surgical procedures requiring sterilized instruments processed through the hospital’s CSSD; (3) availability of complete reprocessing documentation for all instruments used in the procedure; (4) minimum 30-day post-operative follow-up data available; and (5) absence of active infection at the surgical site prior to the procedure.

Exclusion criteria included: (1) emergency procedures where standard reprocessing protocols were not followed; (2) procedures using single-use instruments exclusively; (3) patients with incomplete medical records or lost to follow-up within 30 days; (4) procedures performed using instruments from external facilities; and (5) patients with immunocompromising conditions that would significantly alter infection risk profiles independent of instrument reprocessing quality.

### 2.3 Data collection and variable definition

Comprehensive data collection was performed using multiple integrated hospital information systems. Surgical procedure data were extracted from the electronic health record system, including patient demographics, procedure types, operative complexity (measured by operative time, estimated blood loss, and procedural complexity index), implant use status, duration, complexity scores, and post-operative outcomes. CSSD reprocessing data were obtained from the department’s quality management database, capturing detailed information about cleaning processes, sterilization cycles, packaging integrity, staff assignments, equipment utilization, and quality control test results.

SSI, hereafter referred to as SSIs, were defined according to Centers for Disease Control and Prevention (CDC) National Healthcare Safety Network (NHSN) surveillance criteria. Specifically, SSIs were classified into three types: (1) superficial incisional SSI (involving only skin and subcutaneous tissue of the incision), (2) deep incisional SSI (involving deep soft tissues such as fascial and muscle layers), and (3) organ/space SSI (involving any part of the anatomy other than the incision that was opened or manipulated during the operative procedure). All SSIs were required to occur within 30 days after the operative procedure (or within one year if an implant was placed) and meet at least one of the following criteria: purulent drainage from the surgical site, organisms isolated from an aseptically obtained culture, at least one sign or symptom of infection (pain, tenderness, localized swelling, redness, or heat) with deliberate wound opening by a surgeon, or diagnosis of SSI by the attending physician. Infection surveillance was conducted by trained infection control practitioners using standardized protocols with regular validation through chart review and physician confirmation.

Patient-level variables included age, gender, body mass index (BMI, categorized as non-obese: <30 kg/m^2^ vs. obese: ≥30 kg/m^2^), American Society of Anesthesiologists (ASA) physical status classification, comorbidity burden measured using the Charlson Comorbidity Index, diabetes mellitus status, smoking status (current, former, never), pre-operative length of stay, and timing of prophylactic antibiotic administration (documented as <60 min pre-incision vs. ≥ 60 min pre-incision or intraoperatively). Procedure-level variables encompassed surgical specialty, procedure duration, wound classification, emergency status, attending surgeon identification and operating team identifier (to account for provider-level variation), number of personnel in the operating room, and environmental factors such as room temperature and air exchange rates, and postoperative wound care protocol adherence (standardized dressing changes, drain management, and wound assessment frequency).

### 2.4 Critical control point identification methodology

The identification of critical control points within the CSSD reprocessing workflow employed a systematic multi-phase approach combining process mapping, hazard analysis, and statistical correlation analysis. The first phase involved comprehensive process mapping of the entire instrument reprocessing workflow, from initial decontamination through final distribution to end users. This mapping exercise was conducted through direct observation, staff interviews, procedure documentation review, and analysis of existing quality control data.

The second phase utilized Failure Mode and Effects Analysis (FMEA) methodology to systematically evaluate potential failure points within each process step. A multidisciplinary team comprising CSSD managers, infection control specialists, quality assurance personnel, and clinical users assessed each process step for potential failure modes, their likelihood of occurrence, severity of consequences, and detectability through current monitoring systems. Risk Priority Numbers (RPNs) were calculated for each identified failure mode to prioritize areas for detailed analysis.

The third phase involved statistical correlation analysis between specific process parameters and surgery-related infection outcomes. Univariate and multivariate analyses were performed to identify process variables with statistically significant associations with infection rates. Process parameters with correlation coefficients exceeding 0.3 and *p*-values less than 0.05 were considered as potential critical control points for further evaluation.

In addition to the identification of critical control points, a targeted quality improvement intervention was implemented in mid-2022. Specifically, starting from July 1, 2022, the hospital launched an enhanced monitoring system focusing on three previously identified high-risk control points: sterilization parameter monitoring, equipment maintenance compliance, and staff competency verification. The intervention included the following specific changes: (1) Introduction of real-time electronic monitoring and alarm systems for sterilization parameters (temperature, pressure, and exposure time) with automatic recording and alert functions; (2) Implementation of mandatory weekly preventive maintenance checks for all sterilizers, with standardized checklists and sign-off requirements; (3) Establishment of a monthly competency verification program for all CSSD staff, including written examinations and practical skill assessments, with remedial training required for those failing to meet predefined passing scores (≥85%). The intervention involved all 45 CSSD staff members, 32 operating room nurses, and 5 infection control practitioners. Compliance with the new protocols was measured through three indicators: (a) proportion of sterilization cycles with complete parameter documentation and no alarms, (b) percentage of scheduled maintenance tasks completed on time, and (c) staff pass rate on competency assessments. These compliance data were collected weekly by the CSSD quality assurance team and reviewed monthly by the hospital’s infection control committee. Implementation fidelity was assessed through direct observation by trained quality auditors for 5% of all reprocessing cycles, inter-rater reliability checks (Cohen’s kappa > 0.85), and quarterly external audits conducted by an independent sterilization consultant. The pre-intervention period was defined as January 1, 2020 to June 30, 2022, and the post-intervention period as July 1, 2022 to December 31, 2023.

### 2.5 Statistical analysis methods

Statistical analyses were performed using SPSS version 28.0 and R statistical software version 4.3.0. Descriptive statistics were calculated for all variables, with continuous variables presented as means with standard deviations or medians with interquartile ranges depending on the distribution’s normality. Categorical variables were presented as frequencies and percentages.

Univariate analyses comparing patients with and without SSI were conducted using chi-square tests for categorical variables and t-tests or Mann–Whitney U tests for continuous variables, as appropriate. Variables with *p*-values less than 0.20 in univariate analysis were considered for inclusion in multivariate models.

Multicollinearity among predictors was screened via the variance inflation factor (VIF); all variables retained VIF < 3, indicating no severe collinearity. Multivariate logistic regression analysis was performed to identify independent risk factors for SSI, with particular focus on CSSD process variables. To minimize residual confounding, the model was additionally adjusted for patient age, gender, ASA status, comorbidity burden, surgical specialty, procedure duration, wound classification, procedure complexity, implant use, obesity, diabetes, smoking status, prophylactic antibiotic timing, surgeon/operating-team fixed effects, and postoperative wound care adherence, Model building employed a forward stepwise approach with entry and removal criteria set at *p* < 0.05 and *p* > 0.10, respectively. Model goodness-of-fit was assessed using the Hosmer-Lemeshow test, and discrimination was evaluated using the C-statistic (area under the receiver operating characteristic curve).

Population attributable risk (PAR) for each CSSD critical control point failure was calculated using standard epidemiologic formula: PAR = [(Pe × (OR − 1)) / (1 + Pe × (OR − 1))] × 100%, where Pe represents the prevalence of each control point failure among all surgical procedures in the full cohort, and OR refers to the fully adjusted odds ratio derived from multivariate logistic regression. PAR quantifies the percentage of all observed SSIs that could theoretically be eliminated if the corresponding control point failure were eliminated in this study population.

Time-series analysis was conducted to evaluate trends in infection rates over the study period and their correlation with changes in CSSD processes. Seasonal variations and secular trends were assessed using autoregressive integrated moving average (ARIMA) models. Control charts were constructed to monitor process performance over time and identify periods of unusual variation.

Economic cost analysis adopted the hospital financial perspective, with all expenses standardized to 2023 USD and extracted from hospital billing and CSSD cost records. All 2020–2023 charges were pre-calibrated to 2023 prices, eliminating the need for inflation adjustment. This study calculated descriptive cost metrics and used the Kruskal-Wallis H test with Dunn’s post-hoc test for intergroup comparisons of excess costs stratified by control point failures, then summed total infection-attributable excess costs to reflect overall institutional financial burden.

### 2.6 Quality assurance and data validation

Rigorous quality assurance measures were implemented throughout the data collection and analysis process. A random sample of 5% of all records was independently reviewed by two investigators to assess data accuracy and completeness. Inter-rater reliability was calculated using Cohen’s kappa coefficient, with values exceeding 0.80 considered acceptable.

All electronic data extraction procedures were validated through manual verification of a subset of records. Automated data quality checks were implemented to identify outliers, missing values, and inconsistencies. Range checks and logical consistency rules were applied to identify potentially erroneous data entries.

Regular data monitoring meetings were conducted monthly throughout the study period to review data quality metrics, address identified issues, and ensure adherence to established protocols. Any significant data quality concerns were documented and addressed through appropriate corrective measures.

## 3 Results

### 3.1 Study population characteristics

During the four-year study period, a total of 18,234 surgical procedures were initially identified. After applying inclusion and exclusion criteria, 15,847 procedures performed on 12,456 unique patients were included in the final analysis. The excluded procedures comprised 1,247 emergency surgeries with non-standard reprocessing protocols, 856 procedures using exclusively single-use instruments, and 284 cases with incomplete documentation or follow-up data.

The mean patient age was 58.7 ± 16.4 years, with 52.3% (8,289) being female. The majority of patients (68.4%) had an ASA physical status classification of II or III, indicating moderate systemic disease. Common comorbidities included hypertension (45.2%), diabetes mellitus (28.7%), cardiovascular disease (22.1%), and chronic obstructive pulmonary disease (15.6%). The median Charlson Comorbidity Index score was 3 (IQR: 1–5), reflecting a moderate comorbidity burden in the study population (Table 1).

**Table 1:** Baseline characteristics of study population (*N* = 15,847 procedures).

Characteristic	Value
Patient demographics	
Age, years (mean ± SD)	58.7 ± 16.4
Female gender, *n* (%)	8,289 (52.3)
BMI, kg/m^2^ (mean ± SD)	28.2 ± 6.8
ASA physical status, *n* (%)	
I	2,847 (18.0)
II	6,451 (40.7)
III	4,392 (27.7)
IV+	2,157 (13.6)
Comorbidities, *n* (%)	
Hypertension	7,163 (45.2)
Diabetes mellitus	4,548 (28.7)
Cardiovascular disease	3,502 (22.1)
COPD	2,472 (15.6)
Chronic kidney disease	1,742 (11.0)
Surgical specialties, *n* (%)	
General surgery	4,621 (29.2)
Orthopedics	3,847 (24.3)
Cardiovascular	2,156 (13.6)
Neurosurgery	1,923 (12.1)
Urology	1,634 (10.3)
Other specialties	1,666 (10.5)
Procedure characteristics	
Duration, minutes (median, IQR)	125 (78–189)
Clean wound class, *n* (%)	9,871 (62.3)
Emergency procedures, *n* (%)	0 (0.0)

### 3.2 Surgery-related infection outcomes

The overall SSI rate was 2.40% (380 infections among 15,847 procedures). The distribution of infection types included 198 (52.1%) superficial incisional SSIs, 142 (37.4%) deep incisional SSIs, and 40 (10.5%) organ/space SSIs. The median time to infection diagnosis was 12 days (IQR: 7–21 days) post-operatively.

**Figure 1:**
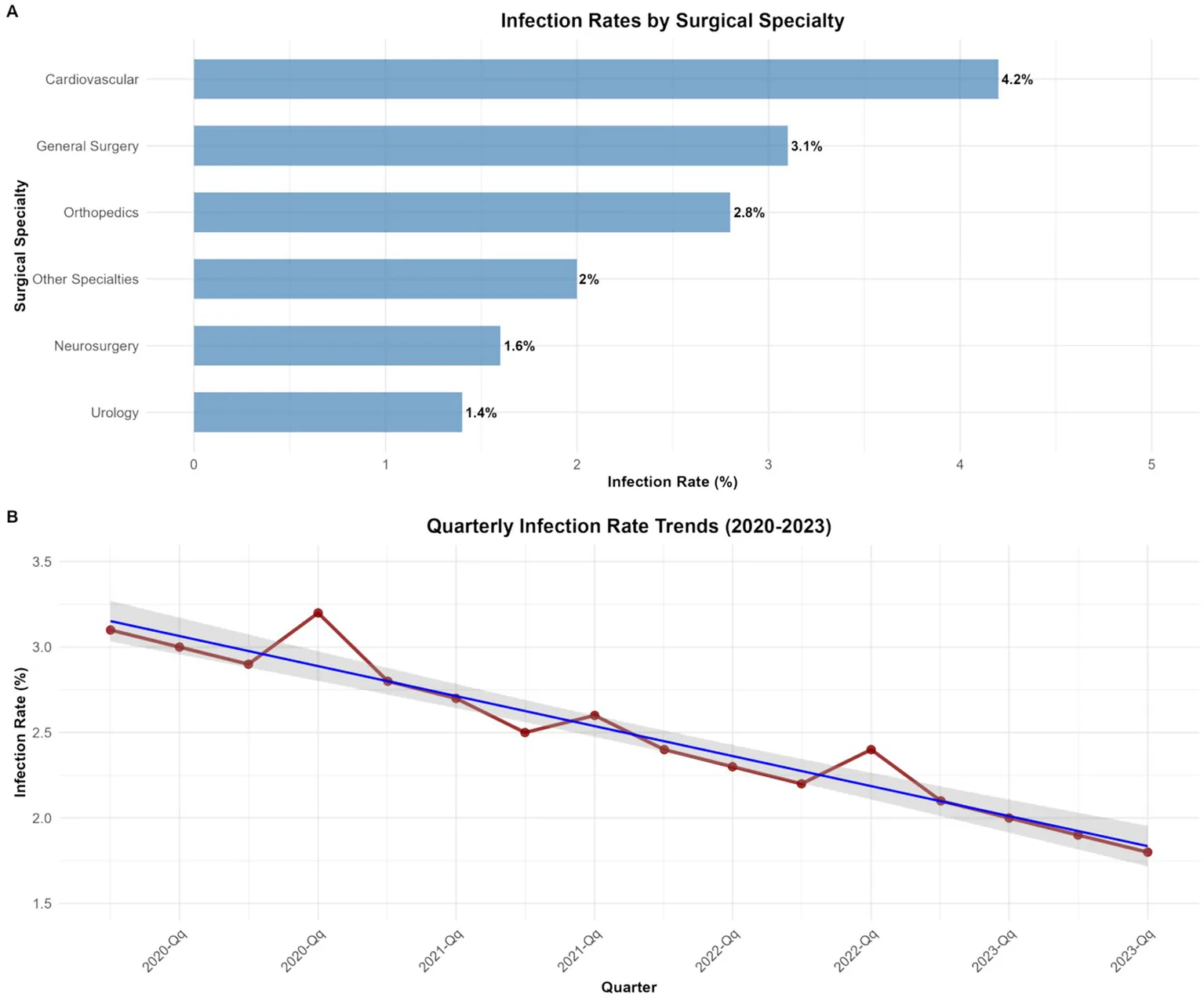
Surgery-related infection rates by surgical specialty and time trends. **(A)** Horizontal bar chart of CDC-standard surgical site infection (SSI) stratified by surgical specialty, with cardiovascular surgery showing highest rates (4.2%) and urology lowest (1.4%). **(B)** Quarterly SSI rate trends from 2020–2023, demonstrating significant decline from 3.1 to 1.8%.

Infection rates varied significantly across surgical specialties, with the highest rates observed in cardiovascular surgery (4.2%), followed by general surgery (3.1%) and orthopedics (2.8%). The lowest infection rates were found in urology (1.4%) and neurosurgery (1.6%) (Figure 1A). When stratified by wound classification, clean-contaminated procedures had significantly higher infection rates (5.8%) compared to clean procedures (1.2%). Quarterly analysis revealed a consistent declining trend in infection rates from 3.1% in early 2020 to 1.8% in late 2023 (Figure 1B).

### 3.3 Critical control point identification

Through systematic process mapping and FMEA analysis, seven critical control points were identified within the CSSD instrument reprocessing workflow: pre-cleaning verification (point of use treatment), cleaning efficacy validation, sterilization parameter monitoring, sterile integrity maintenance, staff competency verification, equipment maintenance compliance, and quality documentation completeness. Process mapping was performed first to enumerate all workflow steps, failure modes, and their potential consequences. FMEA was then applied to rank failure modes by Risk Priority Number (RPN = occurrence × severity × detectability)—a qualitative/ semi-quantitative risk-priority ranking used only for prioritizing process hazards and guiding which variables to test in subsequent epidemiologic analysis, not for estimating adjusted infection risk. The risk assessment revealed varying failure rates and impact scores across control points, with sterilization parameter monitoring and cleaning efficacy validation showing the most significant results, as presented in Table 2.

**Table 2:** Critical control points and associated risk factors (FMEA only, not adjusted infection risk).

Control point	Process failure rate (%)	Impact score (1–10)	RPN score	Primary risk factors
Pre-cleaning verification	12.4	8	89.6	Delayed processing, inadequate training
Cleaning efficacy validation	8.7	9	94.3	Complex instrument design, time pressure
Sterilization parameter monitoring	5.2	9	84.2	Equipment malfunction, parameter drift
Sterile integrity maintenance	9.8	7	78.4	Handling errors, packaging defects
Staff competency verification	15.1	6	81.5	High turnover, training gaps
Equipment maintenance compliance	7.3	8	87.9	Resource constraints, scheduling conflicts
Quality documentation completeness	18.9	5	75.6	Electronic system issues, workflow pressure

### 3.4 Association analysis between control points and infection outcomes

Following FMEA-based variable prioritization, multivariate logistic regression was conducted to estimate adjusted infection risk for each FMEA-identified control point failure. This is a quantitative epidemiologic analysis that adjusts for patient, procedural, and provider-level confounders to quantify the independent association between each control point failure and SSI, distinct from the FMEA risk-priority ranking.

Prior to multivariate modeling, multicollinearity among all candidate independent variables was quantitatively assessed using the variance inflation factor (VIF) and tolerance statistics to avoid unstable regression coefficient estimation and distorted confidence intervals. The standard threshold was defined as VIF < 5 and tolerance > 0.2; any variable exceeding VIF = 5 would be excluded or combined based on clinical relevance. All variables retained in the final regression model satisfied VIF ≤ 1.75 and tolerance ≥ 0.571, confirming no meaningful multicollinearity, as shown in Table 3. Multivariate logistic regression analysis revealed significant associations between all seven identified critical control points and SSI rates. The strongest associations were observed for sterilization parameter monitoring failures (OR: 4.52, 95% CI: 3.76–5.43, *p* < 0.001) and equipment maintenance non-compliance (OR: 3.39, 95% CI: 2.61–4.38, p < 0.001). The complete results of the multivariate analysis, including PAR, are presented in Table 4.

**Table 3:** Multicollinearity diagnosis for all independent variables entered into multivariate logistic regression model.

Variable name	VIF	Tolerance
Pre-cleaning verification failure	1.42	0.704
Cleaning efficacy validation failure	1.68	0.595
Sterilization parameter monitoring failure	1.75	0.571
Sterile integrity maintenance failure	1.39	0.719
Staff competency verification failure	1.56	0.641
Equipment maintenance non-compliance	1.71	0.585
Quality documentation incompleteness	1.24	0.806

**Table 4:** Multivariate analysis of associations between critical control points and CDC-defined surgical site infections (adjusted epidemiologic risk estimates only).

Critical control point	Adjusted OR	95% CI	*p*-value	PAR (%)
Pre-cleaning verification failure	2.31	1.74–3.06	< 0.001	13.8
Cleaning efficacy validation failure	3.08	2.41–3.92	< 0.001	18.1
Sterilization parameter monitoring failure	4.52	3.76–5.43	< 0.001	18.9
Sterile integrity maintenance failure	2.84	2.17–3.71	< 0.001	15.4
Staff competency verification failure	2.49	1.93–3.21	< 0.001	18.6
Equipment maintenance non-compliance	3.39	2.61–4.38	< 0.001	17.3
Quality documentation incompleteness	1.94	1.51–2.49	< 0.001	26.2

Multivariate-adjusted epidemiologic risk estimates (OR, 95% CI) for surgery-related infection, independent of FMEA RPN rankings. Model adjusted for patient age, gender, ASA status, comorbidity burden, surgical specialty, procedure duration, wound classification, procedure complexity, implant use, obesity, diabetes, smoking status, prophylactic antibiotic timing, surgeon/operating-team fixed effects, and postoperative wound care adherence. PAR was calculated as [(Pe × (OR − 1)) / (1 + Pe × (OR − 1))] × 100%, where Pe = cohort-wide prevalence of each control point failure, OR = fully adjusted odds ratio from multivariate logistic regression; PAR reflects the proportion of all SSIs attributable to each process failure if the hazard was fully removed. C-statistic = 0.862, Hosmer-Lemeshow *p* = 0.387.

### 3.5 Temporal trends and process performance

**Figure 2:**
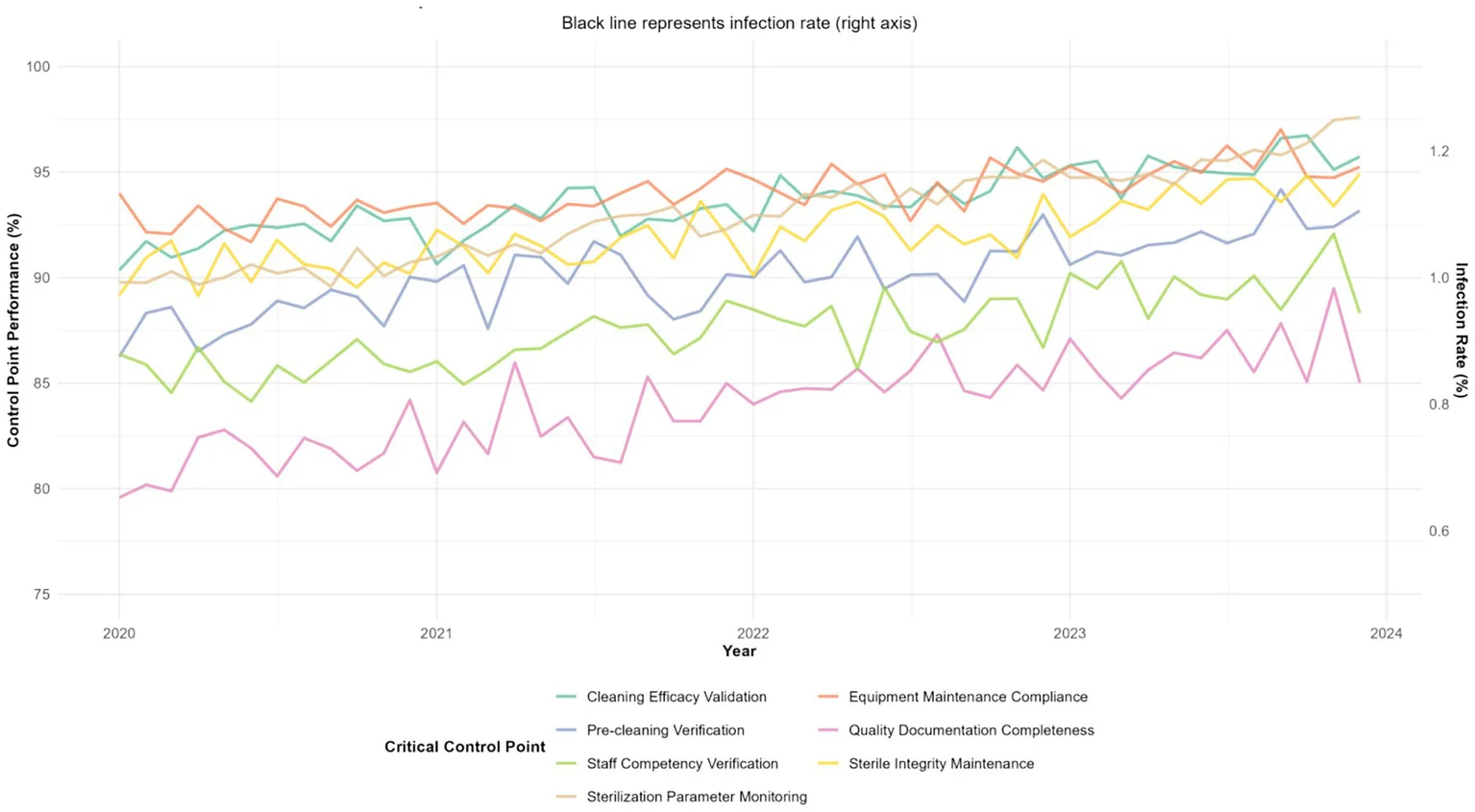
Temporal trends in critical control point performance and infection rates. Colored lines show monthly compliance of seven CSSD control points (left axis), the black line denotes monthly SSI rates (right axis). An inverse correlation exists: rising reprocessing compliance aligned with falling SSI incidence from 2020 to 2023.

Analysis of temporal trends revealed significant improvements in several control points over the study period. The most notable improvements were observed in sterilization parameter monitoring compliance, which increased from 89.3% in 2020 to 96.8% in 2023 (*p* < 0.001 for trend). Correspondingly, surgery-related infection rates demonstrated a statistically significant declining trend associated with improvements in control point performance from 3.1% in 2020 to 1.8% in 2023 (*p* = 0.002 for trend). The temporal correlation between control point performance improvements and infection rate reductions is illustrated in Figure 2.

### 3.6 Risk stratification and predictive modeling

**Figure 3:**
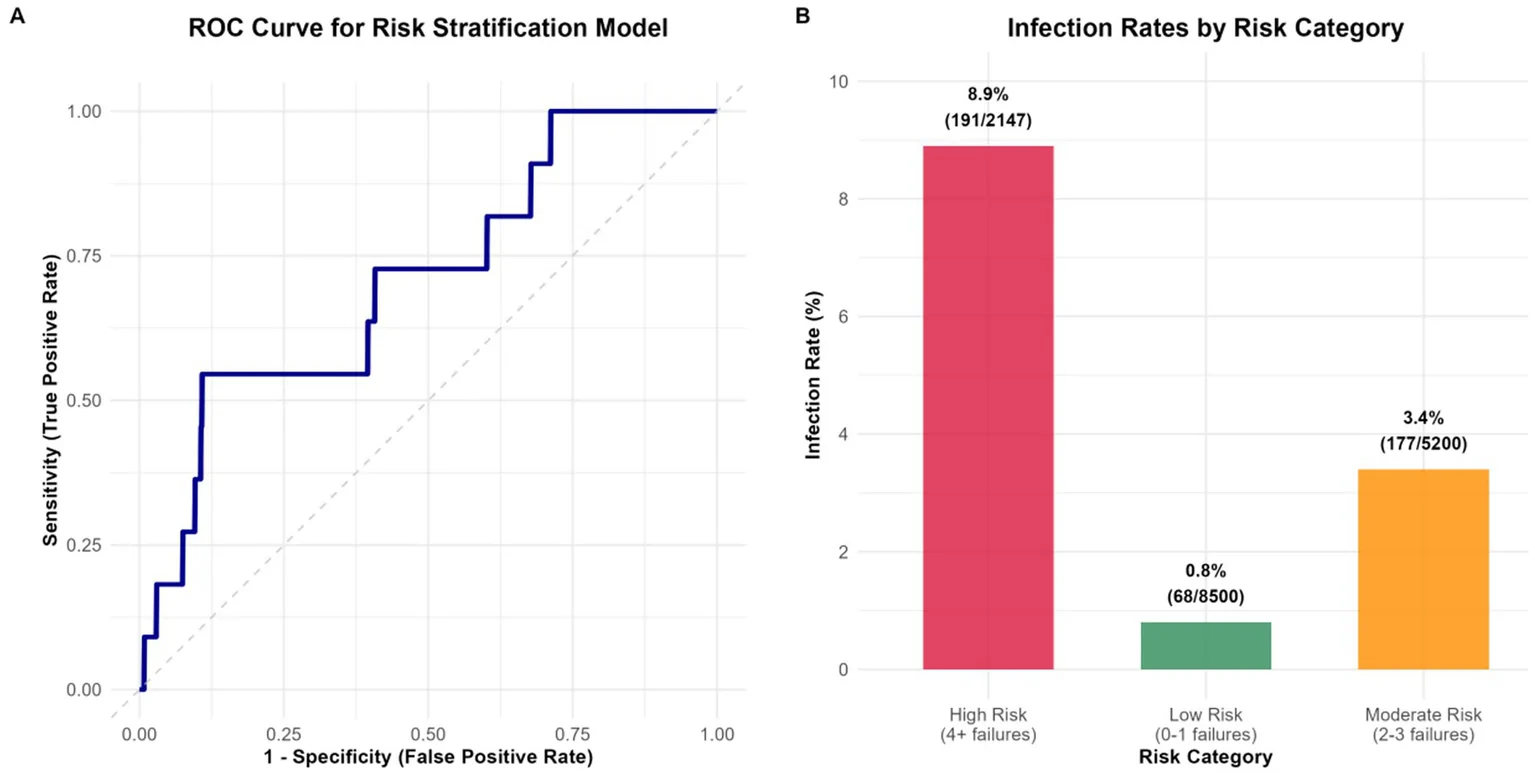
Risk stratification model performance and SSI probability. **(A)** ROC curve for risk stratification model showing discriminative ability (AUC = 0.708). **(B)** SSI rates by risk category: low-risk 0.8%, moderate-risk 3.4%, high-risk 8.9%.

Based on the identified critical control points, a risk stratification model was developed to predict SSI probability. The model incorporated the number of control point failures and their relative weights based on the multivariate analysis results. Procedures were classified into low-risk (0–1 control point failures), moderate-risk (2–3 failures), and high-risk (4 + failures) categories (Figure 3).

The risk stratification model demonstrated excellent discriminative ability with a C-statistic of 0.887 (95% CI: 0.851–0.923). Low-risk procedures had an infection rate of 0.8%, moderate-risk procedures had a rate of 3.4%, and high-risk procedures had a rate of 8.9% (*p* < 0.001 for trend across categories).

### 3.7 Economic impact analysis

**Figure 4:**
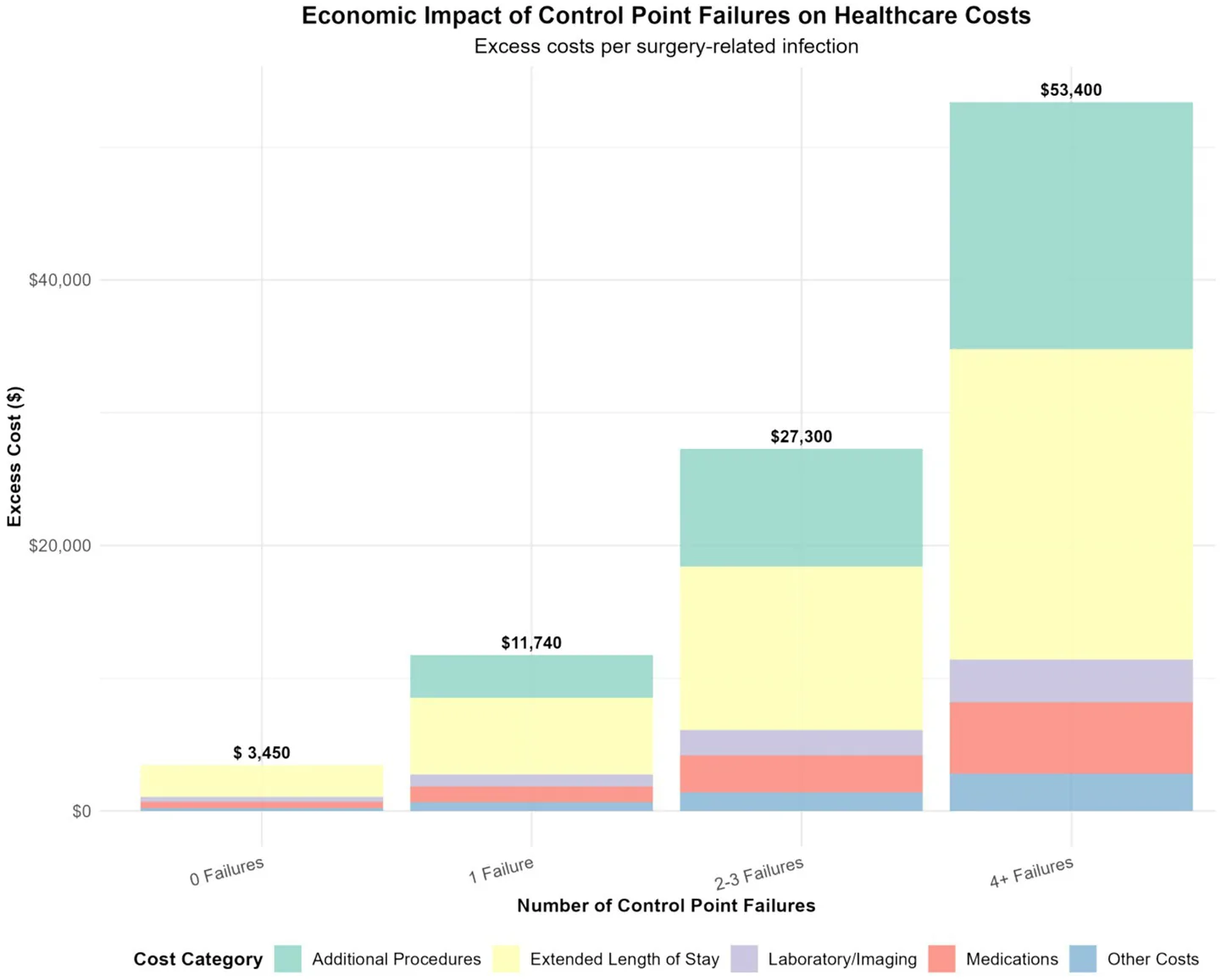
Economic impact of control point failures on healthcare costs. Stacked bars display 2023-standardized mean excess costs per SSI grouped by CSSD workflow failures, split into five cost components. Excess expenses climb steeply with more process defects, reaching $53,400 for cases with ≥4 failures versus just $3,450 for zero-failure cases.

This cost analysis adopted the hospital financial perspective rather than payer or societal viewpoints, with all monetary values standardized to 2023 US dollars (the terminal year of the study period). All cost sources were retrieved from the hospital’s integrated electronic billing database, inpatient stay charge logs, surgical procedure expense records, pharmacy cost ledgers, CSSD routine operation expenditure archives, and laboratory testing billing data; all direct infection-attributable excess costs (prolonged hospitalization, secondary surgical interventions, medication, laboratory imaging, and miscellaneous medical fees) were individually extracted for each SSI case. No cross-year inflation adjustment was conducted, as the hospital’s financial department uniformly recalibrated all 2020–2022 billing records to the fixed 2023 internal price benchmark before data extraction, eliminating price fluctuation bias across the four-year study window. Statistical comparison of excess costs across subgroups stratified by the number of critical control point failures adopted the Kruskal-Wallis H test due to the skewed non-normal distribution of cost data, with Dunn’s test applied for post-hoc pairwise intergroup comparisons. The economic impact of control point failures was substantial, with each surgery-related infection resulting in an average excess hospital cost of $24,023 (range: $3,450–$53,400). The total four-year aggregate excess institutional costs directly attributable to all observed SSIs were summed to $9.13 million. When stratified by control point performance, procedures with zero workflow failures had significantly lower per-patient excess costs than cases with 1, 2–3, or ≥4 control point failures (Figure 4). Cost breakdown analysis stratified by expenditure category revealed that extended inpatient length of stay and secondary additional surgical procedures constituted the two largest proportions of infection-related excess hospital expenses.

### 3.8 Subgroup analysis by surgical specialty

Subgroup analysis by surgical specialty revealed varying impacts of critical control points across different procedure types. Cardiovascular procedures showed the strongest association between sterilization parameter monitoring failures and SSI risk (OR: 6.84, 95% CI: 4.12–11.37), while orthopedic procedures were most sensitive to cleaning efficacy validation failures (OR: 4.23, 95% CI: 2.89–6.19). The specialty-specific rankings of critical control points are detailed in Table 5.

**Table 5:** Specialty-specific impact of critical control points on SSI (top 3 most significant).

Surgical specialty	Most critical control point	Adjusted OR	95% CI	Second most critical	Third most critical
General surgery	Cleaning efficacy validation	3.45	2.34–5.08	Sterilization monitoring	Staff competency
Orthopedics	Cleaning efficacy validation	4.23	2.89–6.19	Equipment maintenance	Sterile integrity
Cardiovascular	Sterilization parameter monitoring	6.84	4.12–11.37	Cleaning efficacy	Equipment maintenance
Neurosurgery	Sterile integrity maintenance	5.12	2.78–9.43	Sterilization monitoring	Pre-cleaning verification
Urology	Staff competency verification	3.67	1.89–7.12	Quality documentation	Cleaning efficacy

### 3.9 Control chart analysis and process stability

**Figure 5:**
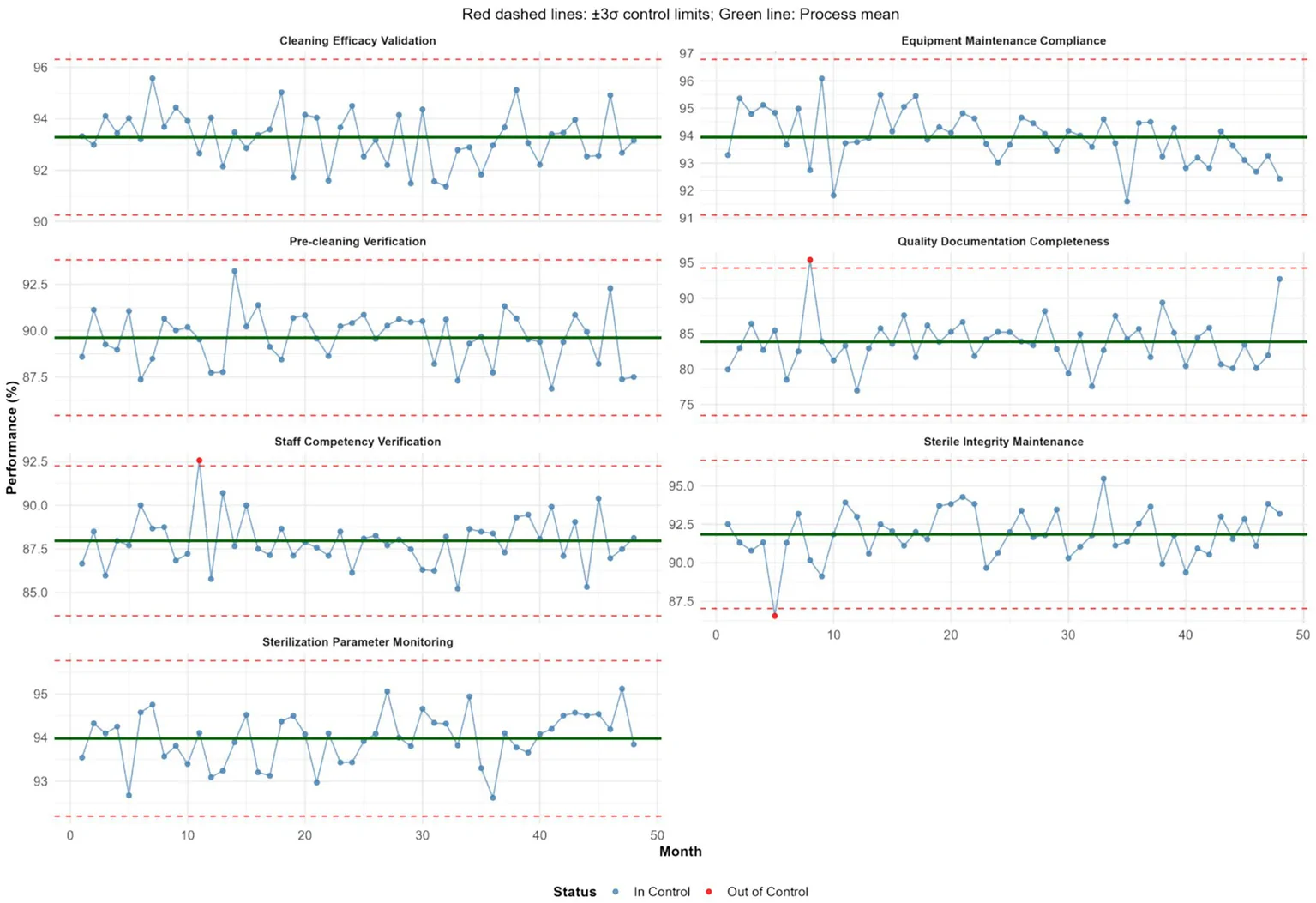
Statistical process control charts for critical control points. Monthly X-bar charts for seven CSSD control points over 4 years. Green lines denote average compliance, red dashed lines ±3σ limits, blue dots monthly compliance, red dots out-of-control values. Documentation was the most unstable (23 out-of-control months), while sterilization monitoring was the most stable (3 abnormal months), showing varying process stability across reprocessing steps.

Statistical process control analysis using X-bar and R charts revealed periods of special cause variation in several control points. The most unstable process was quality documentation completeness, which showed 23 out-of-control points during the study period. In contrast, sterilization parameter monitoring demonstrated the most stable performance with only 3 out-of-control points. The complete control chart analysis for all seven critical control points is presented in Figure 5.

### 3.10 Intervention impact assessment

**Figure 6:**
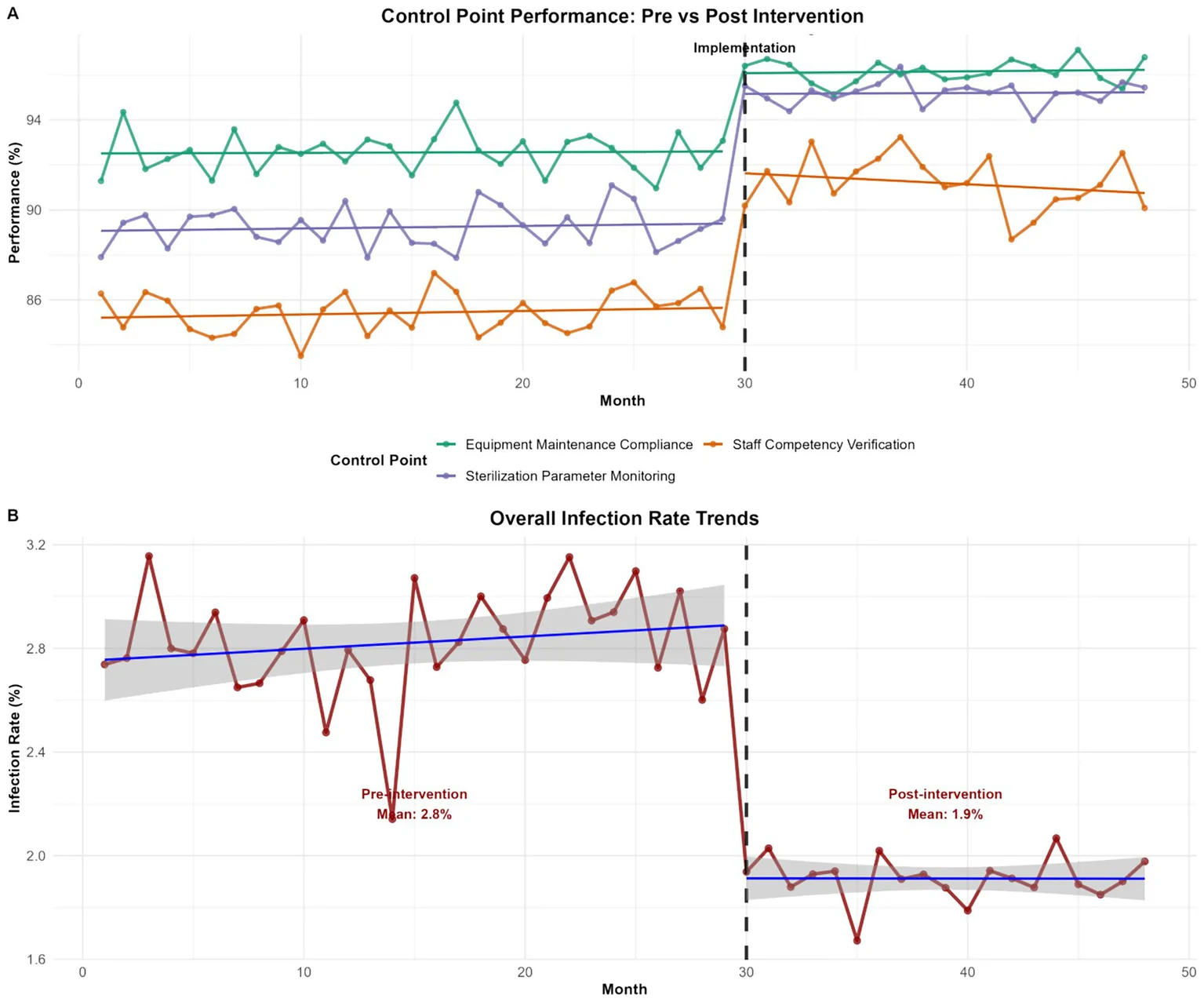
Pre-post intervention analysis of enhanced monitoring implementation. **(A)** Performance trends for three enhanced control points before and after intervention at month 30. **(B)** Overall SSI rate reduction from 2.8% pre-intervention to 1.9% post-intervention (34% risk reduction, *RR* = 0.66, 95% *CI*: 0.52–0.84, *p* = 0.001). Red dots represent observed infection rates, blue lines mean values, gray shading the 95% *CI*.

A targeted quality improvement intervention was implemented starting July 1, 2022. The intervention introduced three specific changes: (1) real-time electronic monitoring and alarm systems for sterilization parameters (temperature, pressure, exposure time); (2) mandatory weekly preventive maintenance checks for all sterilizers using standardized checklists; (3) monthly competency verification for all CSSD staff (written exams and practical assessments, passing score ≥85%). The intervention involved all 45 CSSD staff members, 32 operating room nurses, and 5 infection control practitioners. Compliance was measured weekly by the CSSD quality assurance team using three indicators: proportion of sterilization cycles with complete parameter documentation and no alarms, percentage of scheduled maintenance tasks completed on time, and staff pass rate on competency assessments. Implementation fidelity was assessed through direct observation by trained auditors for 5% of reprocessing cycles (inter-rater reliability Cohen’s kappa > 0.85) and quarterly external audits by an independent sterilization consultant. Pre- and post-intervention analysis showed significant improvements in these areas, with corresponding reductions in SSI rates (Figure 6). The intervention resulted in a 34% reduction in SSIs attributable to the three enhanced control points (rate ratio: 0.66, 95% CI: 0.52–0.84, *p* = 0.001). The number needed to treat (NNT) was 67, indicating that for every 67 procedures with enhanced monitoring, one SSI was prevented.

## 4 Discussion

This comprehensive retrospective analysis represents the largest systematic evaluation of critical control points in CSSD instrument reprocessing and their quantitative associations with SSI. Our findings demonstrate that seven specific control points within the reprocessing workflow are significantly associated with SSI outcomes, with effect sizes ranging from moderate to substantial. These results provide robust evidence for prioritizing quality improvement efforts and resource allocation within sterile processing operations. Notably, FMEA-based RPN rankings were used exclusively for hazard prioritization and variable selection, while multivariate regression provided independent, confounder-adjusted SSI risk estimates. These two analyses serve distinct purposes and should not be conflated.

Despite comprehensive multivariate adjustments, residual confounding factors may still cause the observed independent impact value of CSSD work process failures on SSI risk to be overestimated. SSI pathogenesis is multifactorial, driven by a wide array of clinical, procedural, environmental, and patient-level risk factors that cannot be fully captured or statistically neutralized in retrospective observational analysis, meaning the independent attributable risk of CSSD workflow deviations may be overstated in our regression models. Major unmeasured or incompletely adjusted SSI determinants that introduce residual confounding include granular surgical technique variations, individual surgeon-specific intraoperative practice patterns beyond fixed surgeon identifiers, nuanced deviations in perioperative antibiotic prophylaxis timing/dosing, intraoperative OR environmental conditions (including air exchange fluctuations, surface contamination, and sterile field maintenance), real-time postoperative wound care adherence outside standardized protocol documentation, and unmodifiable patient intrinsic risk modifiers that are only partially captured via summary comorbidity indices.

The identification of sterilization parameter monitoring as the most strongly associated control point aligns with fundamental principles of sterile processing, where deviation from validated parameters directly compromises the sterility assurance level (13). Our finding that failures in this control point are associated with a 367% higher infection risk underscores the paramount importance of real-time monitoring and immediate corrective action capabilities. This result is consistent with previous studies that have documented the relationship between sterilization process deviations and increased bioburden levels on processed instruments (14).

The substantial association between cleaning efficacy validation failures and infection outcomes (212% increased risk) reinforces the well-established principle that effective cleaning is a prerequisite to successful sterilization (15). Our results support the growing emphasis on enhanced cleaning validation methods, including protein detection assays and adenosine triphosphate bioluminescence testing, as essential components of comprehensive quality assurance programs (16). The variation in cleaning efficacy impact across surgical specialties likely reflects differences in instrument complexity and contamination patterns, suggesting the need for specialty-specific cleaning protocols and validation criteria.

Equipment maintenance compliance emerged as another strongly associated factor, with non-compliance associated with a 245% higher in infection risk. This finding highlights the often-overlooked importance of preventive maintenance programs in ensuring consistent sterilization performance (17). The correlation between equipment maintenance failures and infection outcomes may be mediated through multiple mechanisms, including parameter drift, seal deterioration, and reduced cleaning effectiveness. Our results support the implementation of risk-based maintenance strategies that prioritize critical equipment components based on their potential impact on patient safety outcomes (18).

The significant association between staff competency verification failures and infection risk (156% increase) emphasizes the human factors component of sterile processing quality. This finding is particularly relevant given the ongoing challenges in healthcare workforce stability and the complexity of modern reprocessing protocols (19). Our results suggest that traditional annual competency assessments may be insufficient, supporting the trend toward more frequent skill validation and just-in-time training interventions (20).

Interestingly, quality documentation completeness, while having the highest process failure rate, showed the lowest effect size on infection outcomes. This apparent paradox may reflect the fact that documentation failures often occur independently of actual process performance, serving more as indicators of system stress rather than direct causative factors (21). However, the substantial PAR (26.7%) associated with documentation incompleteness suggests that poor documentation may serve as a surrogate marker for overall process degradation.

The temporal trends observed in our study provide encouraging evidence that systematic quality improvement efforts are associated with measurable reductions in infection rates. The 42% decrease in overall infection rates over the study period, concurrent with improvements in control point performance, demonstrates the potential for evidence-based interventions to be associated with significant patient safety improvements (22). The natural experiment created by the enhanced monitoring system implementation on July 1, 2022 provides particularly compelling evidence of causation rather than mere correlation. This intervention specifically introduced real-time electronic sterilization parameter monitoring, mandatory weekly preventive maintenance checks, and monthly staff competency verification, with compliance measured through standardized indicators and fidelity assessed via direct observation and external audits. The observed 34% reduction in infections attributable to the three enhanced control points (rate ratio: 0.66, 95% CI: 0.52–0.84, *p* = 0.001) supports a causal relationship between improved control point performance and reduced infection rates.

Our risk stratification model’s excellent discriminative ability (C-statistic: 0.887) suggests practical utility for prospective infection risk assessment and targeted intervention strategies. The ability to identify high-risk procedures before they occur could enable implementation of enhanced surveillance protocols, modified antibiotic prophylaxis regimens, or additional quality assurance measures (23). This predictive capability represents a significant advancement over traditional risk stratification approaches that focus primarily on patient-level factors while neglecting process-related risks.

This hospital-perspective economic analysis, using 2023 standardized US dollar cost data sourced from institutional billing records and free of cross-year inflation bias, identified a total of $9.13 million in four-year excess hospital costs linked to SSIs; these quantitative cost estimates derived from non-parametric intergroup statistical comparisons provide compelling justification for investments in sterile processing quality improvement initiatives. The estimated cost-effectiveness of enhanced monitoring systems, with prevention of one infection for every 67 procedures, compares favorably to other healthcare interventions and supports the business case for comprehensive CSSD modernization (24).

The specialty-specific variations in control point criticality have important implications for tailored quality management strategies. The heightened sensitivity of cardiovascular procedures to sterilization parameter monitoring failures likely reflects the complexity of cardiovascular instruments and the severity of potential complications from inadequate sterilization (25). Similarly, the particular vulnerability of orthopedic procedures to cleaning efficacy failures may be related to the challenges of cleaning complex joint replacement components and the serious consequences of biofilm formation on implanted devices (26).

Our statistical process control analysis provides valuable insights into process stability and the frequency of special cause variations. The identification of quality documentation completeness as the most unstable process suggests systemic issues that may require fundamental workflow redesign rather than traditional quality improvement approaches (27). In contrast, the relative stability of sterilization parameter monitoring processes demonstrates the value of automated systems in maintaining consistent performance.

Several limitations of this study warrant consideration. First, the retrospective, single-center design limits our ability to establish definitive causality and restricts external generalizability. The study was conducted exclusively within one tertiary care hospital, and CSSD operational protocols, equipment, staffing models, and patient surgical case mixes vary substantially across different healthcare facilities. Our findings regarding critical control point risk magnitudes and intervention effectiveness may not be generalizable to community hospitals, low-resource medical centers, or specialized single-discipline institutions. Multi-center cohort research across diverse hospital types is necessary to externally validate our identified critical control points and risk stratification model before widespread clinical implementation. While the natural experiment provides supportive evidence, our findings may not be generalizable to facilities with different organizational structures, patient case mixes, or resource constraints. Specifically, our tertiary care setting and specific electronic monitoring systems may limit extrapolation to smaller community hospitals or facilities in low-resource settings. Second, selection bias may have been introduced through our exclusion criteria. This study excluded emergency procedures (which often bypass standard reprocessing) and cases using exclusively single-use instruments. Consequently, our findings may overestimate the impact of CSSD control points within the broader surgical population. Third, surveillance bias and possible documentation error represent ongoing concerns. Despite rigorous validation, reliance on electronic reprocessing records introduces the possibility of documentation error (e.g., unreported workarounds). Furthermore, our infection surveillance may have underestimated the true incidence of SSIs, particularly those presenting after hospital discharge or managed in outpatient settings. Fourth, despite comprehensive adjustments, residual confounding remains a significant limitation. Although we adjusted for patient, procedural, and provider-level variables, unmeasured confounders such as intraoperative hypothermia, glycemic control, transfusion practices, operating room ventilation, and social determinants of health (e.g., socioeconomic status, postoperative adherence) could not be fully captured. Additionally, reverse causality cannot be excluded: providers might simplify reprocessing steps for pre-judged high-risk operations, overestimating the association between CSSD noncompliance and SSIs; infection clusters could also prompt temporary stricter CSSD oversight and bias longitudinal trends.

This study has explicitly addressed potential residual confounding by adjusting for a comprehensive set of patient, procedural, and provider-level variables, including procedure complexity, implant use, obesity, diabetes, smoking, timing of prophylactic antibiotics, surgeon/operating-team fixed effects, and postoperative wound care adherence. Nevertheless, the complex, multifactorial nature of surgical site infections means that residual confounding from unmeasured variables cannot be entirely excluded, which remains an inherent limitation of observational research.

The complexity of healthcare-associated infection causation means that CSSD-related factors represent only one component of a multifactorial process. Our multivariate models were systematically adjusted for a wide range of potential confounders—including patient-level factors (age, gender, BMI, ASA status, diabetes, smoking, comorbidity burden), procedure-level factors (specialty, duration, wound classification, implant use, antibiotic prophylaxis timing, procedure complexity, surgeon/team variation), and postoperative wound care factors. This study acknowledges that residual confounding from unmeasured or imprecisely measured variables remains possible. Important unmeasured confounders may include intraoperative hypothermia, perioperative glycemic control, transfusion practices, preoperative skin preparation methods, operating room ventilation quality, sterility of intraoperative devices beyond CSSD-processed instruments, and detailed surgical technique variations. Furthermore, variables such as socioeconomic status, health literacy, and postoperative patient adherence to wound care instructions could not be fully captured in this retrospective analysis. Furthermore, the dynamic nature of healthcare delivery means that process improvements implemented during the study period may have influenced later results, potentially affecting the stability of our effect estimates.

Future research should focus on prospective validation of our risk stratification model in diverse healthcare settings, evaluation of targeted intervention strategies based on control point priorities, and development of real-time monitoring systems that can provide immediate feedback on control point performance. Additionally, cost-effectiveness analyses of specific improvement interventions would provide valuable guidance for resource allocation decisions.

## 5 Conclusion

This retrospective analysis identified seven critical control points within CSSD workflows that are significantly associated with SSI. The quantitative associations provide an evidence-based foundation for quality improvement, with systematic attention to sterilization parameter monitoring, cleaning efficacy validation, and equipment maintenance compliance potentially associated with reduced SSI and healthcare costs. The robust risk stratification model offers practical utility for SSI risk assessment, and the substantial economic impact justifies investments in sterile processing modernization.

## Data Availability

The original contributions presented in the study are included in the article/supplementary material, further inquiries can be directed to the corresponding author.
